# A Legacy of Exposure: Lactational PFAS Transfer and Early-Life Accumulation in Northern Elephant Seals

**DOI:** 10.1021/envhealth.5c00782

**Published:** 2026-06-01

**Authors:** Kara M. Joseph, Sarah J. Rehm, Jessie R. Chappel, Daniel E. Crocker, Rachel R. Holser, Daniel P. Costa, Jane I. Khudyakov, Erin S. Baker

**Affiliations:** † Department of Chemistry, 2331 University of North Carolina at Chapel Hill, Chapel Hill, North Carolina 27514, United States; ‡ Bioinformatics Research Center, Department of Biological Sciences, North Carolina State University, Raleigh, North Carolina 27606, United States; § Department of Biology, Sonoma State University, Rohnert Park, California 94928, United States; ∥ Institute of Marine Sciences, University of California Santa Cruz, Santa Cruz, California 95064, United States; ⊥ Department of Ecology and Evolutionary Biology, University of California Santa Cruz, Santa Cruz, California 95064, United States; # Department of Biological Sciences, University of the Pacific, Stockton, California 95211, United States

**Keywords:** PFAS, maternal transfer, lactation, northern elephant seal

## Abstract

Although PFAS are widely acknowledged as ubiquitous and persistent organic pollutants, critical gaps remain in mapping environmental distribution and clarifying health-relevant exposure pathways, especially in early life. Marine mammal milk provides a unique window into both environmental occurrence and intergenerational transfer, especially in capital-breeding species in which lactation occurs during prolonged fasting with rapid lipid mobilization. Here, we investigated PFAS burdens and maternal transfer in the northern elephant seal (*Mirounga angustirostris*). Elephant seals, with highly characterized nursing behavior and foraging patterns, serve as a valuable sentinel for modeling both patterns of lactational transfer and assessing PFAS concentrations in deep-ocean ecosystems. This study assessed plasma from 14 mother-pup pairs and milk from 9 mothers using a non-targeted data acquisition platform coupling liquid chromatography, ion mobility spectrometry, and high-resolution mass spectrometry (LC-IMS-HRMS). Twenty-seven PFAS were identified across all matrices and age groups, and quantitative analyses revealed concentrations in milk and plasma that exceeded relevant human-based exposure and dietary intake thresholds. Across all pairs, pup plasma PFAS concentrations were consistently higher than maternal concentrations. Specifically, the highest PFAS concentrations were observed in weaned pups (average ∑16 PFAS = 89.08 ng/mL), indicating pronounced early-life accumulation and/or redistribution during the postweaning fast. Concentrations of PFAS in the milk also correlated with maternal and pup plasma levels, demonstrating pair-specific contaminant profiles. Suspect screening further illustrated matrix- and lineage-specific exposure patterns within matched mother-pup pairs, consistent with shared foraging grounds and lactational transfer mechanisms. Together, these findings indicate that neonatal marine mammals in extreme capital-breeding systems experience elevated PFAS body burdens during sensitive developmental windows.

## Graphical abstract

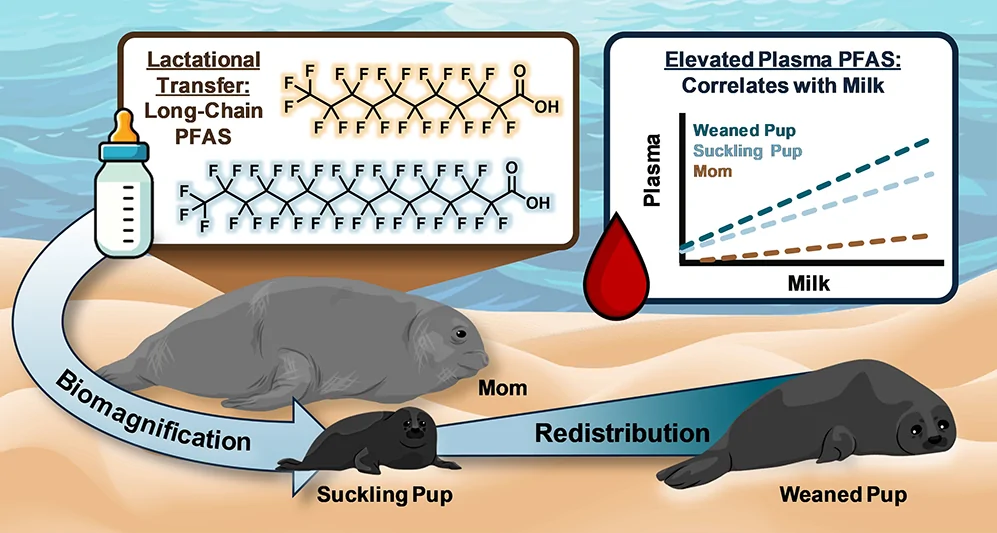

## 1 Introduction

As concern over the ubiquity and rising prevalence of environmental contamination grows, so too does attention on a particular group of compounds: per- and polyfluoroalkyl substances (PFAS). These chemicals in particular are anthropogenic in nature and have been used in a variety of commercial and industrial applications dating back to the 1950s. PFAS are defined by the Organisation for Economic Co-operation and Development (OECD) as any compound containing one fully fluorinated methyl or methylene group.^1,2^ Under this definition, there are over 7 million possible PFAS structures listed in PubChem, and ∼14,000 of these are prioritized for analysis by the United States Environmental Protection Agency (EPA).^3,4^ Many of the PFAS identified by the EPA also meet the criteria as a persistent organic pollutant (POP) under the Stockholm Convention, which includes persisting in the environment, becoming widespread, bioaccumulating, and posing risks to humans and wildlife.^5–9^ Despite the rapidly developing interest in PFAS, many questions remain unanswered. In a recent review, several predominant scientists in the field identified that major gaps in PFAS research lie in identifying their environmental distribution and assessing their health effects.^10,11^ Together, these challenges highlight the need for specific matrices and model systems that link environmental occurrence with meaningful exposure assessments.

Analysis of PFAS in mammalian milk offers a powerful approach for identifying their environmental distribution and elucidating health-relevant exposure pathways. By studying milk, scientists are afforded temporal information regarding the persistence and transfer of PFAS across multiple generations. Furthermore, analyses of milk from aquatic mammals enable insight into the spatial patterns of these chemicals in oceans which are challenging to characterize due to constant circulation and complex ecosystem dynamics. Marine mammals are also accepted as effective sentinels for POP exposure over space and time due to their longevity, position at higher trophic levels, large fat stores that accumulate lipophilic contaminants, and wide-ranging foraging behavior.^12–16^ Northern elephant seals (*Mirounga angustirostris*), in particular, are widely recognized as sentinels for monitoring the pelagic zone due to highly characterized offshore migrations and foraging strategies in the deep, open ocean.^17–19^

One defining biological trait of northern elephant seals is their status as capital breeders. Capital breeding is a life history strategy in which reproduction efforts are fueled primarily by energy reserves accumulated prior to the breeding period rather than by concurrent reliance on dietary intake. This strategy occurs across diverse taxa, including other marine mammals such as gray seals and hooded seals, but reaches an extreme in the northern elephant seals. For instance, in northern elephant seals, females undergo prolonged fasting during lactation (∼4 weeks) while transferring substantial lipid reserves to their pups via energy-dense milk. During this short time, females will lose around 60% of body fat and pups will gain almost 100 kg bodyweight at a rate of ∼4 kg per day, with approximately 55% of this gain comprised of fat, indicative of the rapid transfer of stored resources from mother to offspring.^20^ After pups are weaned, they rely on energy reserves from nursing to support a subsequent 6–8 week period of postnatal development while fasting, which establishes determinants of success within the first year of life.^20–22^ Additionally, lactation in pinnipeds like northern elephant seals differs from other mammals since they do not rely on de novo lipid synthesis, and instead mobilize lipids from internal stores in the blubber to supply milk.^23,24^ This decoupling of feeding from milk production creates a discrete and predictable window of organic contaminant redistribution and transfer from the adipose tissue. For persistent and bioaccumulative compounds such as PFAS, capital breeding may therefore amplify maternal offloading and early-life exposure.

Mother-pup dyads in exclusive capital-breeding species like the northern elephant seal thus provide a powerful system for examining the dynamics of maternal contaminant transfer, biomagnification, and developmental exposure during sensitive life history stages. Previous studies have evaluated brominated and chlorinated POPs in northern elephant seals and concluded that these chemicals increased in the blubber during foraging periods.^25^ Then, during periods of fasting, these lipophilic POPs are mobilized from the blubber to the circulating plasma, which is exacerbated for lactating females and pups during their postweaning fast.^26,27^ Thus, lipophilic compounds like PFAS may follow similar trends, highlighting that nursing and postweaning phases present critical windows for exposure assessments. While previous studies have largely relied on blubber or blood matrices for assessment of legacy hydrophobic compounds, northern elephant seal milk remains an underexplored matrix for investigating the fate and transfer of legacy and emerging PFAS.

In this work, we evaluated plasma samples from 14 mother-pup pairs collected in 2022, with corresponding milk samples from 9 of the females. Using a non-targeted data acquisition platform combining liquid chromatography with ion mobility spectrometry and high-resolution mass spectrometry (LC-IMS-MS), we quantified 29 PFAS in the plasma and milk samples to evaluate how maternal body burdens translate into lactational transfer and early-life accumulation. Quantitative results were complemented by suspect screening, enabling the identification of 10 additional PFAS. The combination of targeted and suspect screening analyses therefore enabled assessments into lineage specificity and correlations between PFAS levels in the milk and plasma of the mothers and pups.

## 2 Materials and Methods

### 2.1 Experimental Design and Sample Information

Northern elephant seal sampling occurred at the rookery in Año Nuevo State Park, California under Marine Fisheries Service Research Permit #23188. This work has been approved by the UC Santa Cruz and Sonoma State University Institutional Animal Care and Use Committees. Samples were collected during another study examining how adult female elephant seals cope with stress challenges and how repeated stress in lactating females may impact pup postnatal development. Aliquots of samples were repurposed for this study to investigate the transgenerational transfer and biomagnification of PFAS in northern elephant seals (Figure 1). Specifically, samples from 14 known-age females with flipper tags applied at weaning and known breeding histories at Año Nuevo were selected for inclusion in this cohort. Adult females were marked with hair dye upon their arrival at the rookery; their pups were also marked with hair dye for identification. More information about mother/pup identification, treatments, and groupings can be found in Table S1. Three weeks postparturition, females were anesthetized using previously described procedures^28^ and blood samples were collected from the extradural vein and placed in chilled ethylenediaminetetraacetic acid (EDTA)-treated vacutainer tubes, consistent with clinical recommendations for blood sampling for PFAS analyses in humans.^29^ Milk samples were collected from 9 of the females as previously described.^30^ Blood samples were also obtained from pups at this time under manual restraint (“suckling pup” group). Approximately 2 weeks later and about 1 week after weaning, a second blood sample was collected from pups under sedation (“weaned pup” group), as previously described.^31^

**Figure 1:**
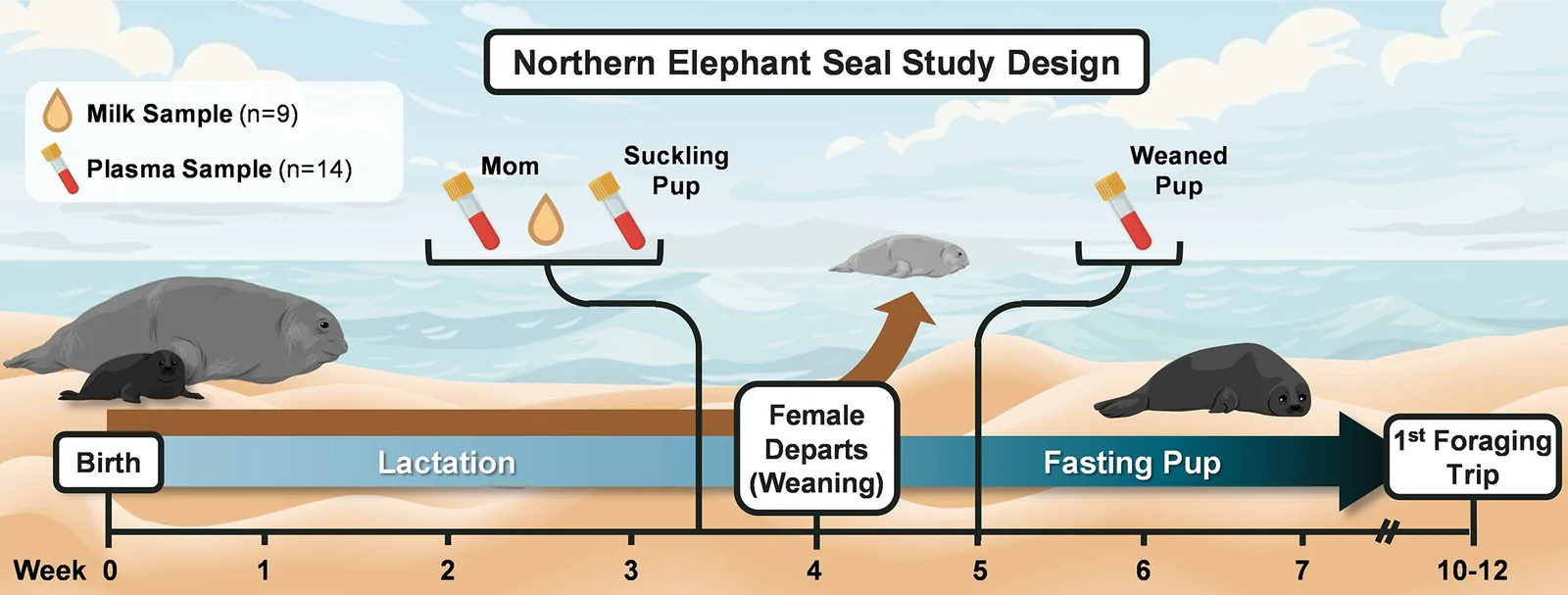
**Experimental Sampling Design.** Elephant seals are born on land. Their mothers fast for the entirety of their 4-week lactation period. Plasma samples were taken from both mother and her suckling pup (n = 14 each), along with a milk sample from 9 of the mothers. After ∼4 weeks, the mothers leave their pups, now considered weaned. A second plasma sample was taken from the pups 1 week after weaning (n = 14). Pups fast for 6–8 weeks following weaning before attempting their first foraging trip in the ocean.

Blood samples were separated by centrifugation for 15 min at 3000 × g at 4 °C and plasma was stored in polypropylene cryovials at −80 °C until further analysis.^32,33^ Milk samples were collected in polypropylene cryovials^33^ and stored on ice until return to the laboratory, at which point they were frozen at −80 °C.

Plasma and milk pools were created to produce quality controls (QCs) by combining equal amounts of each sample, splitting the plasma into their corresponding group (mom, suckling pup, and weaned pup) to create three separate plasma pools and a milk pool.

### 2.2 Standards and Reagents

Native and stable isotope labeled (SIL) standards were purchased from Wellington Laboratories (PFAC30PAR and MPFAC-HIF-ES; Guelph, ON, Canada) and all other chemicals were purchased from Thermo Fisher Scientific (Waltham, MA), unless otherwise specified. Solvents used for sample preparation and instrumental analysis were Optima LC-MS grade unless indicated differently. For catalog numbers and vendor information for all consumables, standards, and reagents, please consult Table S2.

### 2.3 Plasma PFAS Extraction

All elephant seal plasma samples were extracted in a single batch using previously established techniques (Figure S1) following assignment of random coded identifiers to minimize bias during analysis.^34^ Briefly, 50 μL of 0.1% formic acid in water spiked with SIL (2.5 ng/mL) labeled internal standard was added to a 50 μL aliquot of plasma in a 2.0 mL Eppendorf microcentrifuge tube and vortexed. To this, 300 μL of cold acetonitrile was added and vortexed. After a 30 min incubation at −20 °C to induce protein precipitation, samples were centrifuged and a consistent volume of the supernatant transferred to a 1.5 mL microcentrifuge tube. The supernatant was then concentrated to dryness in a SpeedVac at 45 °C and reconstituted in 75 μL of methanol with 4 mM ammonium acetate.

NIST SRM 1950 (Human Plasma) was extracted concurrently using the same procedure, serving as a QC to assess quantification accuracy. An external calibration curve was constructed using charcoal-stripped fetal bovine serum (FBS) spiked with increasing concentrations (final concentrations from 0.001 to 100 ng/mL plasma) of native PFAS standard mix and extracted like all other plasma samples.

### 2.4 Milk PFAS Extraction

All milk samples were extracted using a previously validated adjustment of the FDA’s method for extracting PFAS from food products, C010.03 in a single batch.^35^ The method was adapted to reduce the starting sample volume to 1 mL to accommodate limited elephant seal milk samples, and a full schematic is shown in Figure S2. Specifically, 1 mL of milk was transferred into a 15 mL Falcon tube and its wet weight recorded (in grams). Water (1 mL) and acetonitrile (2 mL) spiked with SIL were added to the tube, along with 20 μL formic acid. Tubes were then vortexed to homogenize. The contents of a QuEChERS salt packet (United Chemical Technologies, Bristol, PA) consisting of 800 mg MgSO_4_ and 200 mg NaCl were added to the tube, which was subsequently shaken using a multitube vortexer (Benchmate VM-MT; Oxford Instruments, Abingdon, United Kingdom) at 2000 rpm for 5 min. Samples were centrifuged at 3900 rpm for 5 min at 4 °C, inducing a phase separation between the organic layer containing lipophilic PFAS and an aqueous layer. An aliquot of the organic layer was transferred to a 2 mL screw-cap dispersive solid-phase extraction (dSPE) tube containing 25 mg primary-secondary amine, 7.5 mg graphitized carbon black, and 150 mg MgSO_4_ (Thermo Fisher Scientific, Waltham, MA) for carbon-clean up. The dSPE tubes were shaken and centrifuged as before. The supernatant was syringe filtered through a 13 mm 0.2 μm pore size nylon Whatman filter. Filtrate was concentrated to dryness using a SpeedVac at 45 °C and samples were reconstituted in 75 μL of methanol with 4 mM ammonium acetate.

NIST SRM 1954 (Fortified Human Breastmilk) was extracted concurrently using the same procedure, serving as a QC to assess quantification consistency and accuracy. An external calibration curve was prepared in commercial goat’s milk (Meyenberg; Turlock, CA). SIL (final concentration 0.5 ng/mL in milk) and increasing concentrations of native PFAS standard mix (final concentrations from 0.005 to 50 ng/mL milk) were added to the acetonitrile, and the calibration curve was extracted like all other milk samples.

### 2.5 Instrumental Analysis

All extracts were transferred to glass LC vials with polypropylene inserts^32^ (Agilent Technologies, Santa Clara, CA) prior to analysis on a platform combining reversed phase liquid chromatography, drift tube ion mobility spectrometry, and high resolution time-of-flight mass spectrometry (RPLC-IMS-HRMS) which has previously been validated for the analysis of PFAS.^34,36–40^ Separations based on polarity were achieved by injecting 4 μL of each sample onto a Zorbax Eclipse Plus C18 column (2.1 × 50 mm, 1.8 μm; Agilent Technologies, Santa Clara, CA) fitted with a corresponding Zorbax Plus C18 guard column (2.1 × 5 mm, 1.8 μm) with the column compartment held at 30 °C and a flow rate of 0.4 mL/min. Mobile phase A was 100% water and mobile phase B was comprised of 95% methanol and 5% water. Both were buffered with 5 mM ammonium acetate. This information, along with the RPLC gradient, can be found in Table S3.

Following LC separation, ions were generated using Agilent’s Jet Stream electrospray source (ESI) (Santa Clara, CA) in negative mode, and introduced to the IMS drift tube with a trap fill time of 3900 μs and a release time of 100 μs.^40^ All analyses utilized 4-bit multiplexing to enhance signal-to-noise ratio.^41,42^ Ultra-High Purity nitrogen was used as the drift tube buffer gas (99.999%; Airgas, Radnor, PA). Data was collected for the 50–1700 *m*/*z* range in MS-only mode using a quadrupole time-of-flight (QTOF) MS set to high sensitivity (2 GHz) mode. All ESI, DT, and MS parameters are summarized in Table S4.

Extracts of the same matrix type were analyzed on the same day in randomized worklists. Since the plasma and milk samples were analyzed at separate time periods, on each day of analysis, ESI-L tune mix (Agilent Technologies, Santa Clara, CA) was flow-injected with identical instrument conditions to ensure accurate mass calibration. These tune files were also used to perform single-field calibration in Agilent MassHunter Workstation Software, IM-MS Browser B.10.00 to obtain accurate and reproducible collision cross section (CCS) values.^43^ Data files were demultiplexed using the PNNL Preprocessor.^41^ All .d files containing retention times (RT), CCS, and *m*/*z* information were annotated using Skyline software, an open-source processing tool, which enables quantitative and suspect screening analyses.^44^ All identifications had RTs within 1.5 min of in-house library standards, a mass error less than 10 ppm and CCS values within the resolving power window of 30.^45^

### 2.6 Quantitative Analysis

Matrix-matched calibration curves were prepared for both plasma and milk as previously stated. Each curve was constructed by calculating the ratio of native-to-heavy peak areas by matching each quantified standard to its corresponding SIL standard. In cases where no SIL match was available in the mixes, a surrogate PFAS with similar headgroup characteristics was assigned (Table S5). The method detection limit (MDL) for each quantified analyte was calculated using a modification on guidance from the EPA (Method 821-R-16-006), based on the concentration of analyte present in the method blank (Figure S3).^46^ The limit of quantification (LOQ) was defined as the lowest point on the calibration curve above the MDL where the relative standard deviation (RSD) of injection replicates was less than 20%.^47^ All calibration curves were constructed with 1/(x^2^) regression weighting and resulted in R^2^ ≥ 0.95. MDLs and LOQs are listed in Table S5 and all identifications are noted in Table S6. Additionally, calculated concentrations of PFAS in the NIST SRMs were used to assess quantitation accuracy (Figure S4) with all values within ±30% the reported value.

### 2.7 Suspect Screening Analysis

Skyline software enables the identification of analytes beyond those in the calibration curves through comparison to an in-house library.^44^ In this study, a library of 156 PFAS with RT, CCS, and *m*/*z* values constructed using certified standards, was used for suspect screening.^48^ As these analytes were not quantified and therefore not included in the calibration curve, the MDL was defined based on the raw peak area of the method blanks, rather than calculated concentrations (Figure S3). The 10 identified PFAS are listed in Table S6.

### 2.8 Statistical Analysis, Data Cleaning, and Transformation

All statistical analyses were conducted using R (version 4.5.1) in RStudio (version 2025.09.1 + 401). To eliminate skew in the data for analytes that were either below the MDL or between the MDL and the LOQ, all plasma and milk concentrations were imputed. This also removes NA values. For more details on this process please see Figure S5. Associations between PFAS concentrations and maternal adiposity, parturition, and age were evaluated, but no substantial correlations were identified (Figure S6).

#### 2.8.1 Paired Plasma-PFAS Concentration Comparisons

Prior to univariate testing, principal component analysis (PCA) was performed on log_2_-transformed imputed individual PFAS concentrations to assess multivariate differences among biological groups (mom, suckling pups, and weaned pups). Then, for each biological sample, individual imputed PFAS concentrations were summed across analytes to generate a total PFAS concentration per replicate. To evaluate differences in summed PFAS concentrations among biological groups, a nonparametric Friedman test was applied. The Friedman test was selected because the data represented repeated measures within matched mother-pup pairs. Furthermore, the selection was due to the repeated measures violating the assumption of independence required for parametric tests, and because Shapiro-Wilk indicated departures from normality despite log_2_ transformation, which was successful in eliminating extreme outliers. In this framework, each life history group was treated as the within-subject factor and each pair was treated as the blocking variable. When applied, the Friedman test indicated a significant overall effect. Thus, posthoc pairwise comparisons between groups were performed using Wilcoxon signed-rank tests with paired observations. To control for multiple comparisons, p-values were adjusted using the Benjamini–Hochberg (BH) false discovery rate correction. Statistical significance was defined as an adjusted p-value (p_adj_) ≤ 0.05. In addition to summed PFAS, groupwise comparisons were computed using the same statistical test for each individual PFAS to assess compound-specific patterns across mothers, suckling pups, and weaned pups.

#### 2.8.2 Milk-PFAS Correlations

An omnibus multivariate analysis of variance (MANOVA) was used to evaluate whether PFAS concentrations in milk (continuous predictor) were associated with PFAS concentrations in plasma for each group (dependent variables: moms, suckling pups, and weaned pups). Prior to conducting the test, assumptions were assessed. Multivariate normality of residuals was evaluated using the Henze-Zirkler test, and bivariate scatter plot matrices were constructed to confirm approximately linear relationships between predictor and outcome variables. Because the predictor is continuous (rather than defining discrete groups), a formal test of equality of covariance matrices (e.g., Box’s M) was not appropriate for this model. Therefore, Pillai’s trace was selected for inference, as it is generally more robust (less sensitive) to violations of covariance homogeneity than Wilks’ Lambda. Although correlations among the dependent variables were extremely high (r > 0.9), the outcomes were analyzed jointly to account for their strong interrelationship and to provide a single overall test of association with milk PFAS. Follow-up univariate ANOVAs were then conducted for each plasma outcome to identify which groups showed individual associations with PFAS in milk.

## 3 Results and Discussion

### 3.1 Elevated Long-Chain PFAS

To quantify the extent of PFAS exposure in northern elephant seals, we first measured plasma concentrations for the mothers, suckling pups, and weaned pups since it is a standard matrix for evaluating internal dose. Of the 29 targeted PFAS, 17 were detected above our platform’s limit of detection and 16 were quantified above the limit of quantification (LOQ) in at least one of the 42 plasma samples (Table S7). We then summed the 16 above the LOQ (∑16 PFAS) to assess a total quantifiable PFAS, which averaged 56.43 ± 36.20 ng/mL for all animals and ranged from 6.13 to 149.28 ng/mL. This sum was dominated by perfluorooctanesulfonic acid (PFOS), perfluorononanoic acid (PFNA), perfluorodecanoic acid (PFDA), and perfluoroundecanoic acid (PFUdA), all of which are long-chained, legacy PFAS as defined by the OECD.^2^ The predominance of long-chain PFAS is consistent with their environmental persistence. Furthermore, they are known to bioaccumulate and biomagnify in marine food webs, leading to higher internal burdens in apex predators such as pinnipeds.^49–51^ This trend is likely exacerbated by the northern elephant seals’ foraging grounds in the mesopelagic zone, which is hypothesized to be a hotspot for long-chain PFAS accumulation.^52^

To place these plasma concentrations in an ecological context, we compared the northern elephant seal PFAS burdens to those reported in other pinnipeds and marine apex predators. Routti et al. measured PFAS in blood plasma of adult lactating Weddell seals from McMurdo Sound, Antarctica, a comparatively remote system, and reported PFUdA at 0.08–0.23 ng/mL, whereas the northern elephant seal plasma PFUdA values averaged 16.43 ng/mL (∼70–200× higher).^53^ Elevated long-chain PFAA burdens in upper-trophic-level marine mammals have likewise been reported in more industrialized or source-influenced regions, including high PFOS and long-chain PFCA concentrations in Arctic polar bear plasma, and high PFOS concentrations in ringed seal blood from the Baltic Sea.^54,55^ This further suggests how proximity to populated coastlines can strongly influence PFAS burdens at the top of marine food webs, while highlighting that these trophic levels are dominated by long-chain PFAS accumulation.

### 3.2 PFAS Biomagnification in Pups

To evaluate whether plasma PFAS burdens differed among mothers, suckling pups, and weaned pups, we imputed values below reporting limits and log_2_-transformed concentrations before performing multivariate analysis. PCA, an unsupervised multivariate dimensionality-reduction technique, was then applied to assess whether overall PFAS profiles differed among groups. PCA (PC2 vs PC1, Figure S7) revealed a strong life-stage pattern with mothers clustered apart from pups. Within pups, separation between suckling and weaned individuals was also apparent, consistent with additional shifts in PFAS profiles across the weaning transition, best visualized with PC3 vs PC1 (Figure 2A). These group-level differences were also reflected in summed concentrations as mothers had a mean ∑16 PFAS of 17.90 ng/mL, whereas suckling pups averaged 62.33 ng/mL (>3× higher than mothers) and weaned pups averaged 89.08 ng/mL (≈5× higher than mothers). This further illustrated that the highest plasma burdens occur in pups and intensify after weaning (Figure 2B and C).

**Figure 2:**
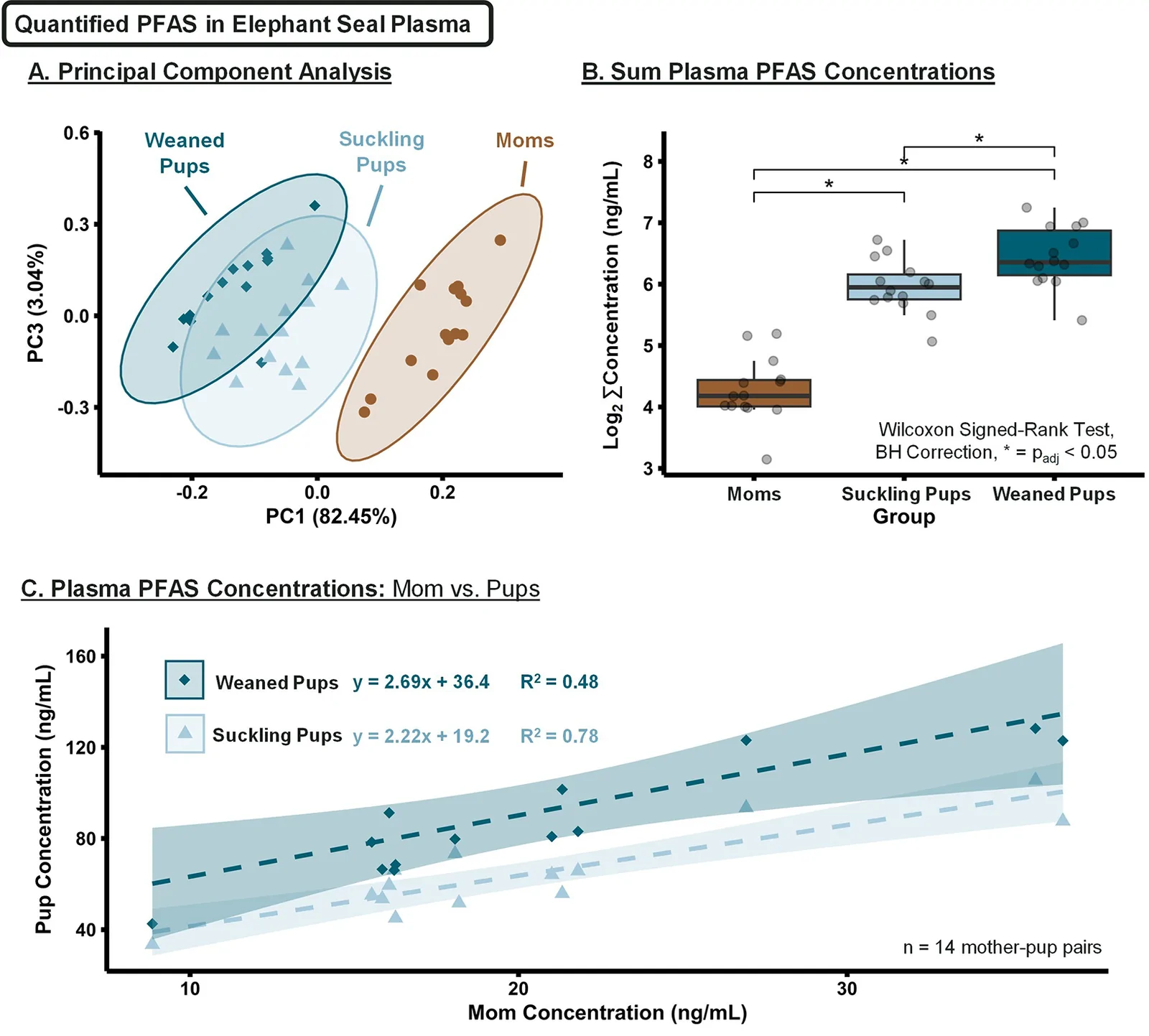
**Quantified PFAS in Elephant Seal Plasma**. (A) Principal component analysis of log_2_-transformed imputed plasma concentrations of 16 PFAS (n = 14 individuals per group). (B) Boxplot showing the log_2_-transformed Σ16 PFAS levels in plasma. Pairwise comparisons were computed with Friedman tests, followed by Wilcoxon Ranked-Sign Test, with * indicating Benjamini-Hochberg corrected p-values less than 0.05 (n = 14 pairs). (C) Linear regression of log_2_-transformed Σ16 PFAS in pup vs mom plasma (n = 14 pairs). Regression analysis shows suckling pup plasma concentrations are more correlated to mom concentrations (y = 2.22x + 19.3, R^2^ = 0.78) compared to weaned pups (y = 2.69x + 36.4, R^2^ = 0.48).

To contextualize northern elephant seal plasma PFAS concentrations using a familiar human biomonitoring framework, we compared results to clinician follow-up guidance developed for humans by the National Academies (NASEM).^56^ NASEM recommends interpreting plasma using the simple additive sum of seven PFAS (Σ7 PFAS = MeFOSAA + PFHxS + PFDA + PFUdA + PFOS + PFNA). Concentrations <2 ng/mL for Σ7 PFAS are considered a range where PFAS-related health effects are not expected. Σ7 PFAS levels between 2 and <20 ng/mL indicate potential for adverse effects, especially for neonates and nursing women, and values ≥20 ng/mL indicate increased risk for related complications.^56^ In our study, Σ7 PFAS ranged from 3.55–21.07 ng/mL in adult females, 18.57–65.55 ng/mL in suckling pups, and 22.78–90.54 ng/mL in weaned pups. Notably, all samples exceeded 2 ng/mL and the proportion exceeding 20 ng/mL increased sharply in pups: 14% of mothers, 92% of suckling pups, and 100% of weaned pups. Although these thresholds were developed for human clinical follow-up rather than wildlife toxicity, the comparison emphasizes that northern elephant seals experience high internal PFAS burdens and that the exposure is markedly elevated in pups during the first 4 weeks of life and into the postweaning period.

To formally test whether plasma PFAS concentrations differed across life stages within matched mother-pup dyads, we next conducted comparisons of plasma PFAS concentrations among mothers and pups at the two different time points. Friedman tests with subsequent Wilcoxon signed-rank tests revealed that across the dyads, the sum PFAS concentrations increased significantly from mothers to suckling pups and again as the pups were weaned (p_adj(BH)_ < 0.05 for all pairwise comparisons, Figure 2B). This pattern was also consistent across individual PFAS, with all of the 16 quantified analytes exhibiting the same increasing trend (Figure S8). Because pups consistently carried higher PFAS burdens than their mothers, we also evaluated the extent to which pup plasma concentrations were associated with maternal plasma concentrations within pairs. Plasma PFAS concentrations in pups were positively associated with maternal plasma concentrations at both time points (Figure 2C). The maternal plasma concentrations explained a substantial proportion of variability in suckling pup plasma concentrations (R^2^ = 0.78), whereas the association was weaker, but still evident after weaning (R^2^ = 0.48) The stronger association during suckling is consistent with closer coupling between maternal and pup PFAS burdens early in life, while the reduced explanatory power after weaning suggests the influence of additional factors.

The life-stage patterns observed by the PFAS plasma concentrations may, in part, reflect how nutrient transfer and physiological redistribution shape northern elephant seal PFAS burdens during early development. During the suckling period, mothers rapidly mobilize large stores of energy and nutrients—and associated contaminants, including PFAS—into milk, which can amplify pup contaminant concentrations relative to mothers.^20^ After weaning, however, pups undergo rapid changes in body composition as they rely on their own energy reserves,^21^ redistributing nutrients (and potentially PFAS) from the blubber to circulating plasma, altering PFAS concentrations independent of maternal plasma. Consistent with this mechanism, previous studies of northern elephant seal paired tissue-plasma matrices show that the postweaning fast promotes blubber-to-blood redistribution of trace elements and organochlorine pollutants.^27,57,58^ Accordingly, such maternal offloading during lactation and pup redistribution during the postweaning fast processes could contribute to the increases in PFAS observed across early-life stages.

### 3.3 Lactation: A Major Source of Early-Life Exposure

To directly assess the potential contribution of lactation to pup exposure, we next characterized PFAS concentrations in northern elephant seal milk collected from 9 of the mothers concurrent with plasma sampling. We targeted 29 PFAS for quantification in milk. Of these, 12 were detected above the LOQ in at least one sample (Table S8). Across these 12 analytes, (∑12 PFAS), total milk concentrations averaged 5.875 ± 2.065 ng/mL and ranged from 2.948 to 8.561 ng/mL. Importantly, these concentrations should be interpreted in the context of the northern elephant seal capital-breeding strategy: females deliver large volumes of energy-dense milk that exceed immediate pup caloric requirements to rapidly build lipid reserves that support the prolonged postweaning fast. One study calculated that, on average, northern elephant seal pups consumed 137.7 kg of milk over the 4-week lactation period.^59^ Using this value and the average milk density of this study (Table S8), we can estimate that the pups would consume over 144,000 mL of milk in total (Figure S9). Multiplying this intake by the average Σ12 PFAS concentration yields an estimated cumulative dietary exposure of ∼848,000 ng of PFAS during lactation, and this total may exceed 1 mg for pups nursing from more highly exposed females (Figure S9). Thus, even though the absolute concentrations of PFAS in milk were lower than those observed in plasma, the volume of milk consumed over a short lactation window indicates that milk can represent a substantial early-life exposure pathway. While there are currently no toxicity or health-based guidance values for milk, since it accounts for all the food consumed by pups during suckling, we can instead utilize dietary intake recommendations to establish approximate safe consumable levels of PFAS. The European Food Safety Authority (EFSA) establishes a recommendation for dietary tolerable weekly intake (TWI) of the sum of four PFAS (Σ4 PFAS = PFOS + PFHxS + PFOA + PFNA) at 4.4 ng/kg bw/week in humans based on epidemiological studies identifying immunotoxic effects during early development.^60^ Using allometric scaling and the average bodyweight of suckling pups whose milk is analyzed here, we translate this TWI to a value of 4.0 ng/kg bw/week for the northern elephant seals (Figure S9).^61,62^ Based on the estimated intake calculations described previously, we therefore approximate that the weekly intake for the Σ4 PFAS for the northern elephant seals during lactation was 676 ng/kg bw/week, or almost 170× what would be considered “safe” under the scaled TWI threshold (Figure S9). Thus, these results highlight lactation as a major and potentially consequential source of early-life PFAS exposure in northern elephant seals.

Following a deeper analysis of the individual PFAS transferred during lactation, the milk samples were predominantly comprised of PFUdA, PFNA, PFDA, PFOS, and perfluorotridecanoic acid (PFTrDA) (Figure 3A). These are all long-chain PFAS, which have also been detected at high concentrations in dolphin milk.^63^ Additionally, four of these PFAS were the main drivers of total PFAS in the northern elephant seal plasma, suggesting that milk concentrations may track maternal and pup plasma burdens. To statistically evaluate this relationship, we fit an omnibus MANOVA with the milk Σ12 PFAS concentration as a continuous predictor and plasma Σ16 PFAS concentrations in mothers, suckling pups, and weaned pups as multivariate outcomes. The model indicated a significant overall association between milk and plasma PFAS concentrations (Pillai’s trace = 0.85, F­(3,5) = 9.75, p = 0.016). Follow-up univariate regressions showed that the milk Σ12 PFAS levels significantly predicted plasma Σ16 PFAS in mothers (F­(1,7) = 16.33, p = 0.0049), suckling pups (F­(1,7) = 9.64, p = 0.017), and weaned pups (F­(1,7) = 24.64, p = 0.0016) (Figure 3B). Similar trends were also observed for each of the 12 PFAS taken individually (Figure S10). Together, these results indicate that higher milk PFAS burdens are associated with elevated plasma burdens in both mother and pups, with the strongest association for weaned pups. This pattern may reflect cumulative PFAS accumulation over the lactation period. As pups begin to mobilize lipid reserves during the postweaning fast, PFAS acquired during lactation may be redistributed into circulation, strengthening the milk–plasma correspondence by the time weaned pups were sampled. In contrast, suckling pup plasma concentrations appear more tightly coupled to maternal plasma, consistent with closer physiological coupling during active nursing and/or a greater relative contribution of prenatal (placental) exposure early in life.

**Figure 3:**
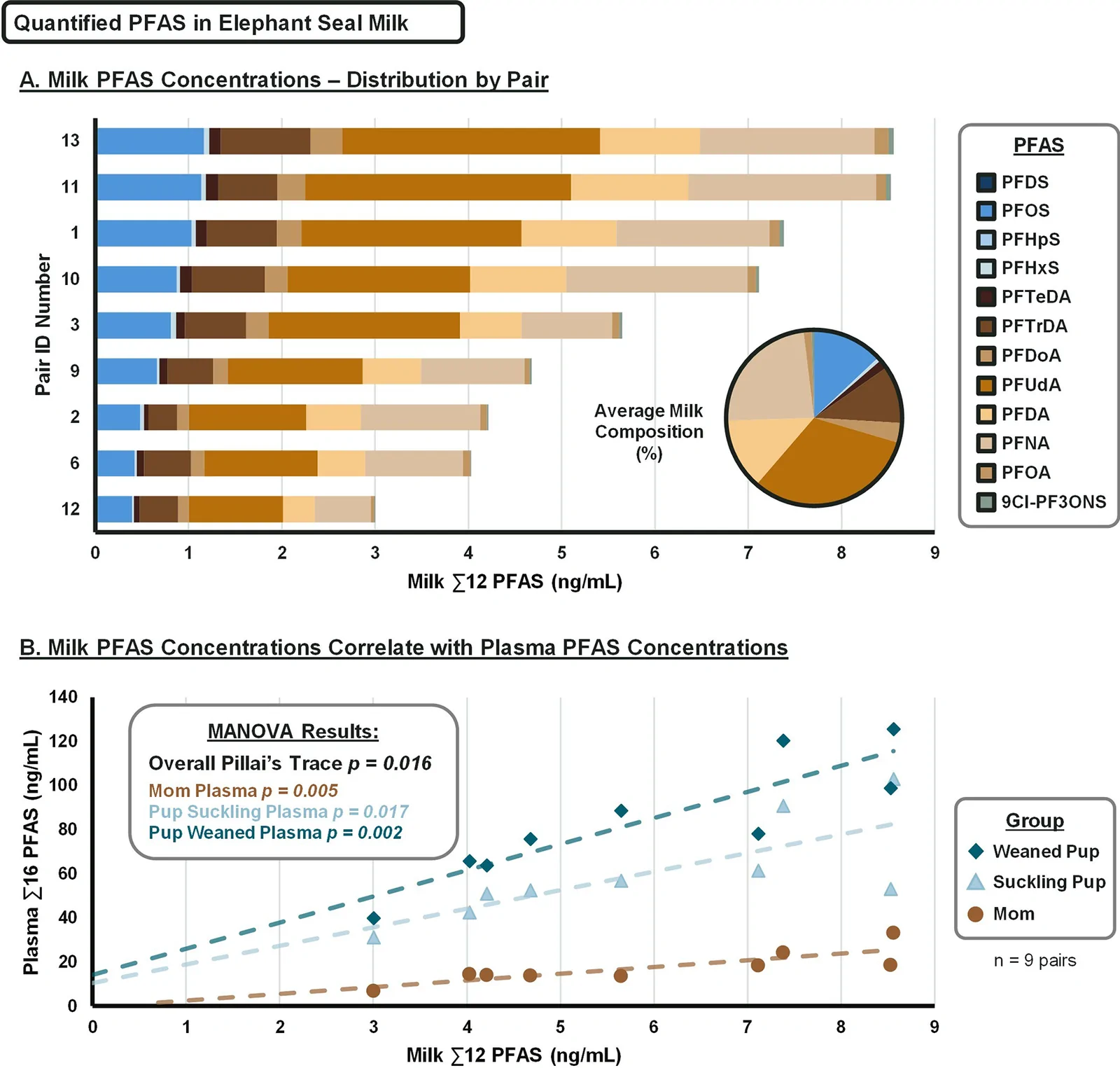
**Quantified PFAS in Elephant Seal Milk**. (A) Stacked bar chart of the Σ12 PFAS (ng/mL) values contributing to overall concentration in milk samples and corresponding to each dam-pup pair. The pie chart shows the average percent contribution of each PFAS to the total concentration. (B) Σ16 PFAS quantified in the dam, suckling pup, and weaned pup plasma plotted against the corresponding Σ12 PFAS quantified in the milk corresponding to that pair (n = 9 pairs). MANOVA results show a significant association between milk and plasma concentrations (Pillai’s trace = 0.85, F­(3,5) = 9.75, p = 0.016). The Σ12 PFAS milk levels significantly predicted plasma Σ16 PFAS concentrations in mothers (F­(1,7) = 16.33, p = 0.0049), suckling pups (F­(1,7) = 9.64, p = 0.017), and weaned pups (F­(1,7) = 24.64, p = 0.0016).

To assess whether the summed PFAS trends reflected consistent patterns within individual mother-pup pairs, we examined trajectory plots for each PFAS commonly quantified between both matrices (Figure 4). Across each PFAS, each pair showed the same progression, with concentrations lowest in milk, higher in maternal plasma, and further elevated in pup plasma both during suckling and after weaning. This consistency across samples suggests that while individual variation is present, the northern elephant seals at this site have comparable PFAS exposure patterns, reflecting their common use of pelagic, deepwater foraging grounds that are far removed from major contaminant sources. It is also possible that these shared trends are indicative of a conserved pathway for the metabolic redistribution and maternal transfer of PFAS in this species of pinniped, but other age classes of northern elephant seals, such as juveniles and adult males (which forage at distinct locations from adult females) would need to be assessed to determine this.^64^

**Figure 4:**
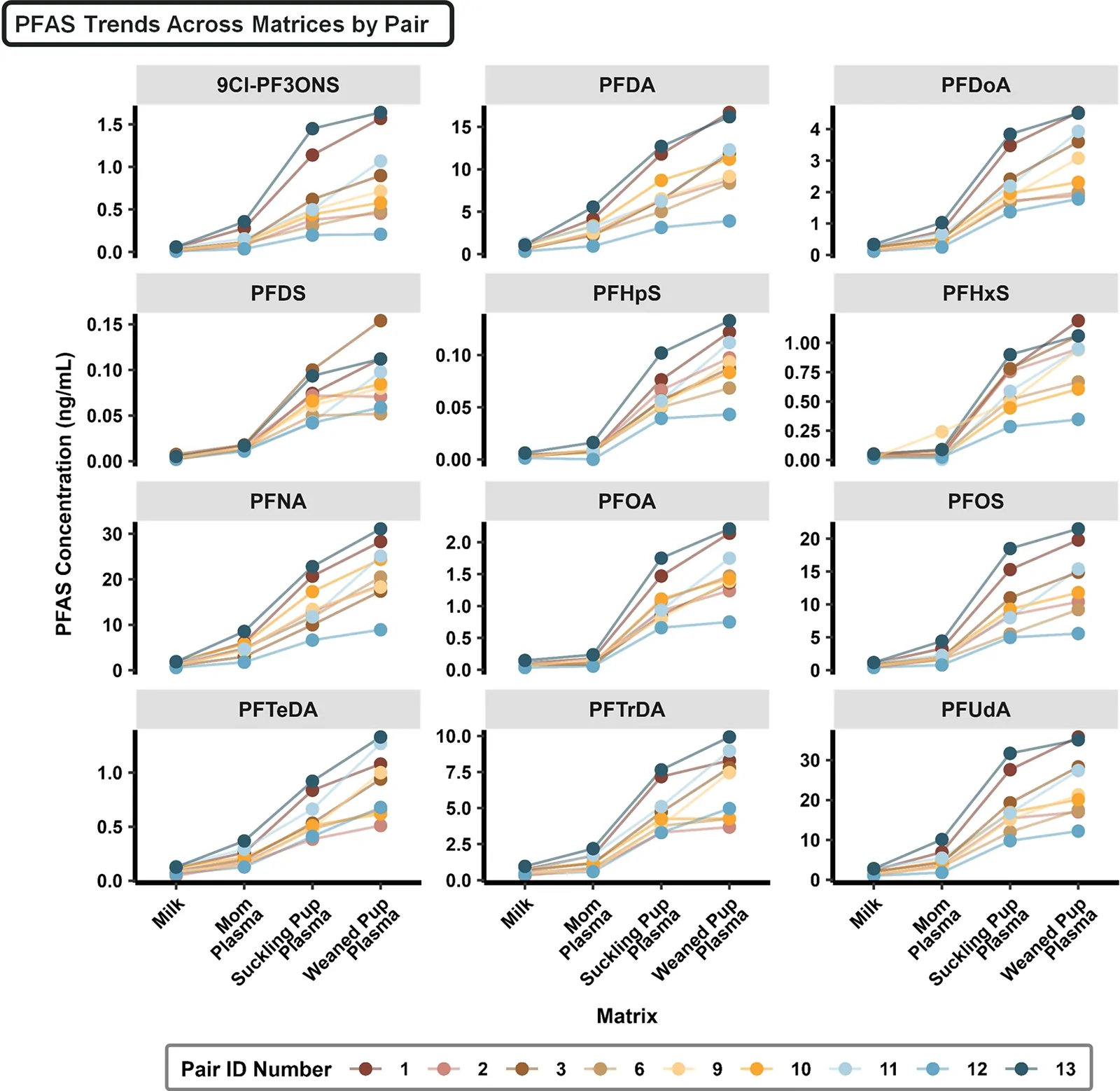
**PFAS Trends Across Matrices by Pair.** Trajectory plots demonstrate trends in increasing concentrations (ng/mL) from milk to mom’s plasma to suckling pup plasma to weaned pup plasma are consistent across PFAS species, while indicating some individual variation across each dyad, represented by Pair ID Number (n = 9 pairs).

### 3.4 Suspect Screening

Although the quantitative PFAS data highlight similarities in northern elephant seal exposure, this represents only a small subset of the most commonly detected PFAS. To complement these targeted analyses, we also performed suspect screening to search for an additional 126 emerging PFAS. In all the plasma and milk samples, an additional 10 PFAS were identified (Figure S11), including analytes from the fluorotelomer, phosphinic acid, sulfonic acid, carboxylic acid, and ether carboxylic acid classes. An intersection analysis was conducted to assess the distribution of these analytes across each mother-pup pair, as well as their commonality between matrices. To evaluate PFAS overlap an UpSet plot was used, where intersection bar heights represent the number of pairs in which a given PFAS was detected (Figure 5A). In this representation, a single PFAS can contribute multiple counts to an intersection if it is detected in multiple mother–pup pairs, such that higher bars reflect both broader matrix overlap and greater consistency across pairs. Consequently, PFAS that appear in intersections spanning many combinations of matrices represent compounds that are widely distributed across life history stages. Conversely, PFAS contributing to low-count intersections reflect compounds detected in only a small number of pairs or matrices and may represent low-abundance or sporadic exposures. In plasma, several PFAS were detected across maternal, suckling pup, and weaned pup plasma in many pairs; for example, 6:6, 6:8, and 8:8 perfluorophosphinic acids (PFPi) co-occur across all three plasma matrices in nine pairs. In contrast, some analytes were detected only in pup plasma. 6:8 PFPi occurred in four pairs in pups but not mothers, and 8:8 PFPi and perfluorohexadecanoic acid (PFHxDA) were detected only in weaned pups.

**Figure 5:**
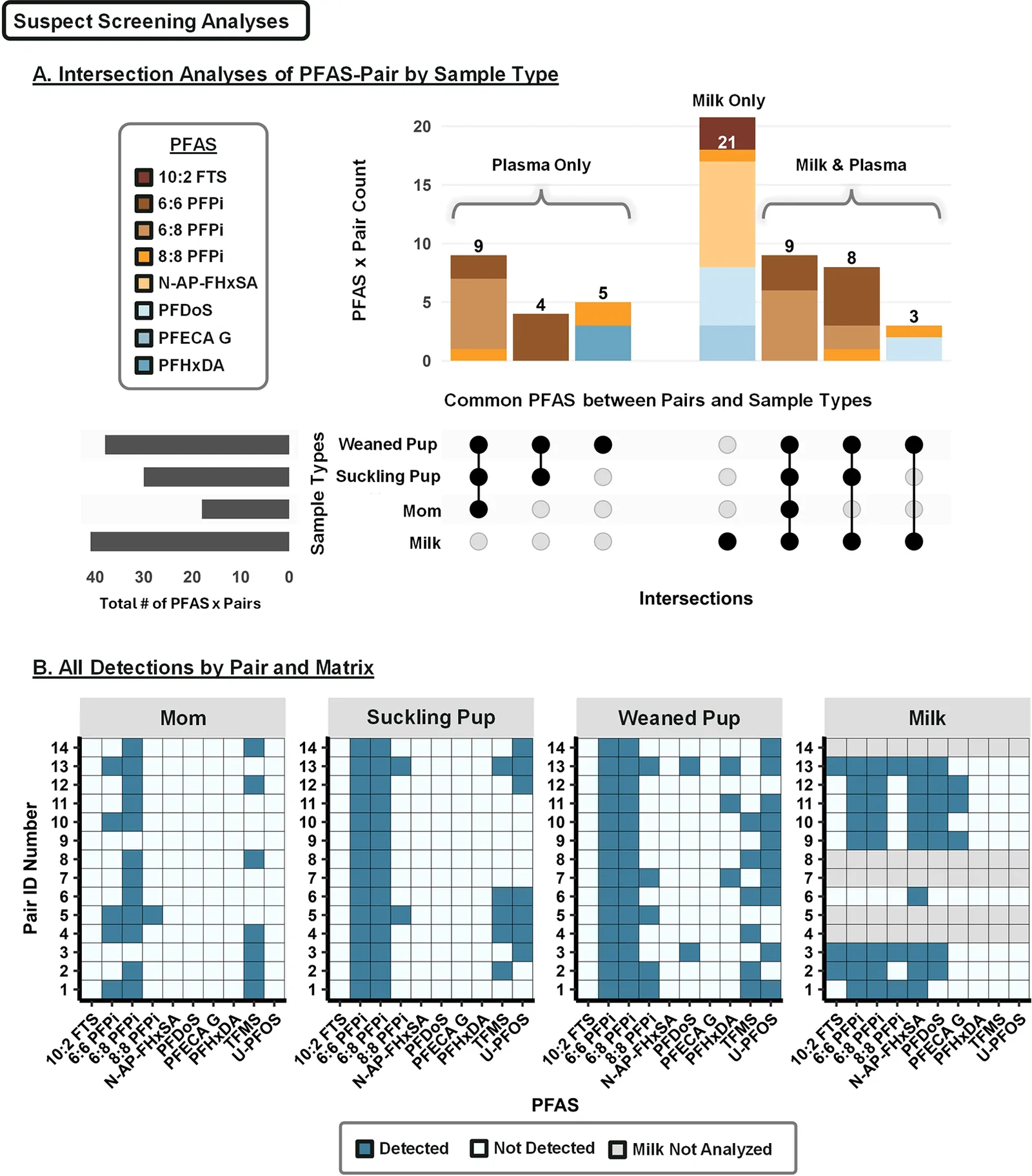
**Suspect Screening Analysis Results.** (A) UpSet plot showing overlap in PFAS detection across biological matrices and life stages, including milk, maternal plasma, suckling pup plasma, and weaned pup plasma. Intersections indicate PFAS detected above reporting limits in the indicated matrix combinations, and bar heights represent the number of dam–pup pairs in which each PFAS was detected for a given intersection. (B) Heatmap displaying patterns in suspect screening PFAS detections across each matrix for all pair numbers (n = 14 pairs).

The largest intersection set in this study corresponded to PFAS detected only in milk, indicating that milk can reveal exposures that are not fully captured by plasma measurements alone. For example, milk was the only matrix in which 10:2 fluorotelomer sulfonate (10:2 FTS) was detected, which may reflect greater analytical recovery in milk and/or limited persistence in circulating plasma. In addition, perfluorododecanesulfonic acid (PFDoS) and perfluoro-4-isopropoxybutanoic acid (PFECA-G) were detected only in milk and in weaned pup plasma, suggesting that these compounds are more readily observed in milk and may only exceed reporting limits in plasma when concentrations are high. Milk may therefore be an important matrix to consider as a complement to plasma analyses when considering neonatal exposure.

Suspect screening analyses can also be used to identify pair-specific exposures to environmental PFAS profiles through a process known as fingerprinting. In previous studies, researchers have used unique PFAS identifications or isomer distributions to indicate point-sources of contamination.^65–67^ We propose that PFAS unique to certain mother-pup pairs could be used to identify generational lineage or migratory foraging patterns, especially when coupled with satellite tagging. This technique has previously been implemented for analysis of mercury through the mesopelagic food web.^19^ The pair-specific suspect screening identifications across each sampled matrix are shown in Figure 5B with some PFAS only seen in specific mothers and their pups. While this study lacks sample size, migratory information, or emission data necessary to draw such conclusions, we see this as a viable and innovative method to both assess environmental distribution and biological accumulation of PFAS.

### 3.5 Potential Implications of PFAS Exposure

Although the effects of high PFAS concentrations in plasma or exposure through lactation have not been evaluated for northern elephant seals specifically, several studies of other marine mammals have seen potential adverse health impacts. In other marine apex predators, elevated PFAS burdens have also been linked to altered immune function and endocrine disruption. For instance, a 2013 study found positive associations between chronic PFAS exposure and several markers of immune, hematopoietic, kidney and liver dysregulation in bottlenose dolphins.^68^ Highly exposed sea otters were found to be more susceptible to common infectious diseases and in polar bears, elevated tissue and circulating concentrations of PFAS were linked to alterations of thyroid homeostasis, neurological implications, and hormone imbalances.^69–72^ Furthermore, a study found that the physiological, endocrine, and reproductive effects reported in humans were similar to those observed in polar bears.^73^ An in vitro study using cDNA from Baikal seals, another capital-breeding pinniped, found that PFNA and PFDA, two PFAS that were prominent in this study, disrupted PPARα–CYP4A signaling pathways in the liver.^74^ Because this pathway is central to lipid metabolism, these disruptions may be particularly relevant during periods of fasting and rapid lipid mobilization.^75,76^ In humans, PFAS exposure has been consistently associated with alterations in energy storage-related lipids, triglycerides and phosphatidylethanolamines, and elevated cholesterol, further suggesting disruption to hepatic lipid metabolism.^77–80^ Taken together, these wildlife studies, alongside human biomonitoring evidence, support the notion that elevated PFAS exposure could pose health risks for marine mammals, particularly during sensitive stages of growth and development.

## 4 Conclusions

In this study, we assess PFAS detected in the plasma of mother-pup pairs at two different time points to understand maternal transfer. Milk collected during the first time point was also evaluated to understand how PFAS were transferred from moms to pups. A total of 17 PFAS were identified in plasma and 12 in milk via targeted analysis, and another 10 PFAS were revealed through suspect screening techniques. PFAS plasma concentrations increased significantly in the pups with mothers averaging 17.89 ng/mL total PFAS, compared with 62.33 ng/mL in suckling pups and 89.08 ng/mL in weaned pups. All plasma samples analyzed in this study exceeded human-derived biomonitoring thresholds and nearly all pup plasma concentrations fell within ranges considered of high concern for PFAS-associated outcomes, including kidney cancer, thyroid disease, dyslipidemia, liver complications and decreased immune response.^56^ Although these thresholds were developed for humans, evidence from marine mammals has reported associations between PFAS exposure and similar physiological end points.^68,70,72,74^

By incorporating the analyses of milk with traditionally utilized plasma measurements for biomonitoring, we provide a complementary matrix which demonstrates trends consistent with rapid maternal contaminant transfer and physiological redistribution of PFAS during early development. This monitoring technique may be a less invasive way to screen for early-life PFAS exposure, eliminating the need for neonatal blood sampling. Furthermore, these results highlight that milk consumption represents a major pathway of early-life PFAS exposure and is exacerbated when species like northern elephant seals rely on capital breeding strategies.

It is important to note that this study is not without its limitations. These samples only represent a small cohort of northern elephant seals from a single rookery, and while the consistencies observed across mother-pup pairs broadly suggests common exposures, geographic differences in foraging and breeding locations may variably influence PFAS profiles and concentrations in other populations. Despite these limitations, this work provides clear evidence that lactation represents a critical and understudied pathway for PFAS exposure and accumulation early in life. Overall, the results highlight how legacy PFAS continue to shape exposure trajectories across generations, especially in apex predators like marine mammals. In this sense, the patterns observed in northern elephant seals reflect a broader legacy of PFAS contamination, one in which persistent chemicals are transferred, retained, and amplified during early development, and whose implications require further attention and research.

## Supplementary Material





## Notes

Figure S1: plasma PFAS extraction schematic; Figure S2: milk PFAS extraction schematic; Figure S3: definitions of MDL and LOQ for quantitative and suspect screening analyses; Figure S4: determination of quantification accuracy to NIST SRMs; Figure S5: quantification imputation procedure; Figure S6: correlations between plasma and selected variables; Figure S7: additional plasma PCA information; Figure S8: pairwise comparisons of plasma concentrations across groups per PFAS; Figure S9: example of dietary intake estimate calculations; Figure S10: plasma vs milk concentrations by PFAS; Figure S11: full suspect screening intersection analysis (PDF)
Table S1: elephant seal sampling information; Table S2: chemicals, equipment, and consumables; Table S3: RPLC information; Table S4: instrumental information; Table S5: targeted analytes, internal standards, MDL, & LOQ; Table S6: all identified PFAS; Table S7: quantified PFAS in plasma; Table S8: quantified PFAS in milk (XLSX)
