# Supplementary material for: A Legacy of Exposure: Lactational PFAS Transfer and Early-Life Accumulation in Northern Elephant Seals
Source: Environ Health (Wash). 2026 Jun 1;4(9):2093–105. doi: 10.1021/envhealth.5c00782 (PMC13595381; doi:10.1021/envhealth.5c00782)

Supporting Information

"A Legacy of Exposure: Lactational PFAS Transfer and Early-Life Accumulation in Northern Elephant Seals"

Kara M. Joseph[1], Sarah J. Rehm[1], Jessie R. Chappel[2], Daniel E. Crocker[3], Rachel R. Holser[4,5], Daniel P. Costa[4,5], Jane I. Khudyakov[6]*, Erin S. Baker[1]*

[1]Department of Chemistry, University of North Carolina at Chapel Hill, Chapel Hill, NC 27514, United States

[2]Bioinformatics Research Center, Department of Biological Sciences, North Carolina State University, Raleigh NC 27606, United States

[3]Department of Biology, Sonoma State University, Rohnert Park, California, 94928, United States

[4]Institute of Marine Sciences, University of California Santa Cruz, California, 95064, United States.

[5]Department of Ecology and Evolutionary Biology, University of California Santa Cruz, California, 95064, United States.

[6]Department of Biological Sciences, University of the Pacific, Stockton, California, 95211, United States.

*Corresponding Author Emails: erinmsb@unc.edu & jkhudyakov@pacific.edu

Table of Contents

Cover page and table of contents **S1-2**

Figure S1. Plasma PFAS Extraction Schematic **S3**

Figure S2. Milk PFAS Extraction Schematic **S4**

Figure S3. Definitions of MDL and LOQ for Quantitative and Suspect Screening Analyses **S5**

Figure S4. Determination of Quantification Accuracy to NIST SRMs **S6**

Figure S5. Quantification Imputation Procedure **S7**

Figure S6. Correlations between Plasma and Selected Variables **S8**

Figure S7. Additional Plasma PCA Information **S9**

Figure S8. Pairwise Comparisons of Plasma Concentrations Across Groups per PFAS **S10**

Figure S9. Example of Dietary Intake Estimate Calculations **S11**

Figure S10. Plasma vs. Milk Concentrations by PFAS **S12**

Figure S11. Full Suspect Screening Intersection Analysis **S13**

Figure S1. Plasma PFAS Extraction Schematic

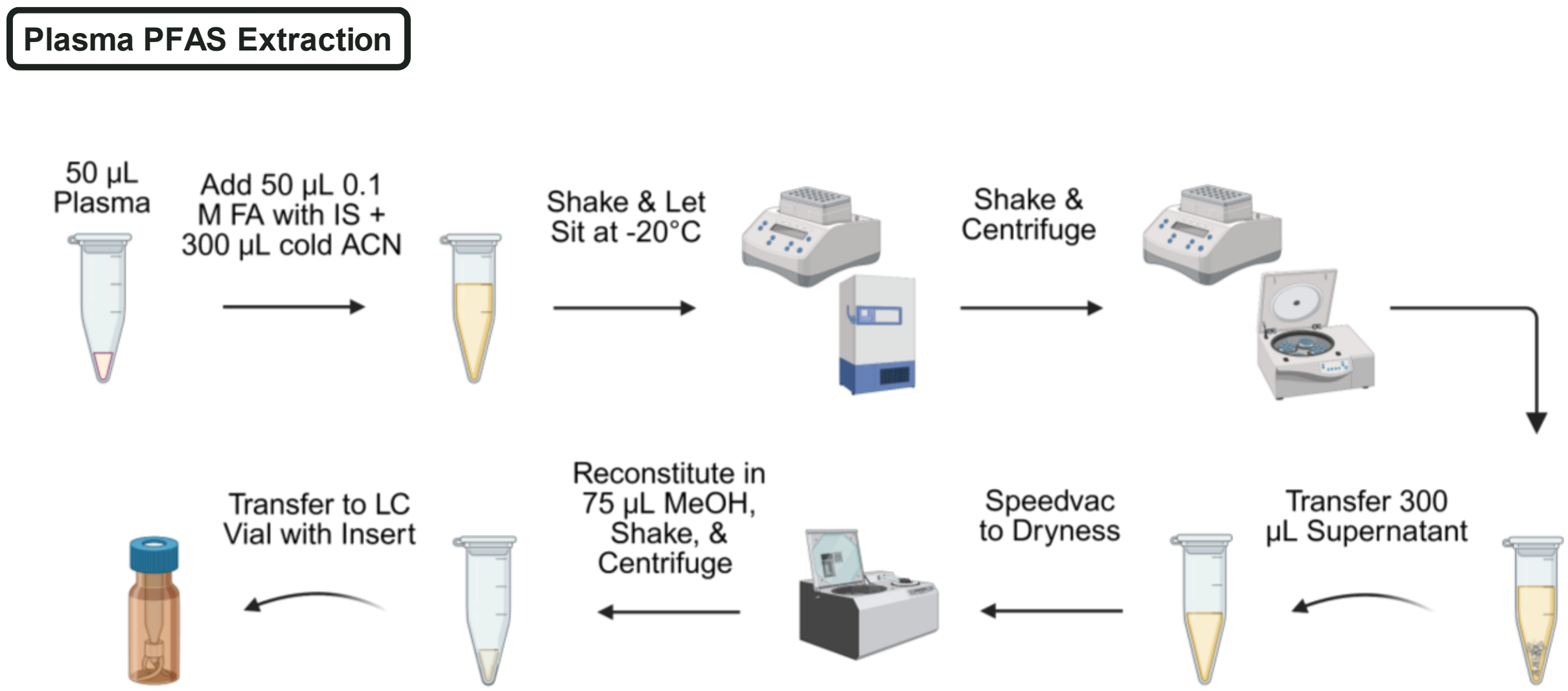

FA = Formic Acid, IS = ^{13}C-Labeled PFAS Mix (MPFAC-HIF-ES), ACN = Acetonitrile, MeOH = Methanol

Figure S2. Milk PFAS Extraction Schematic

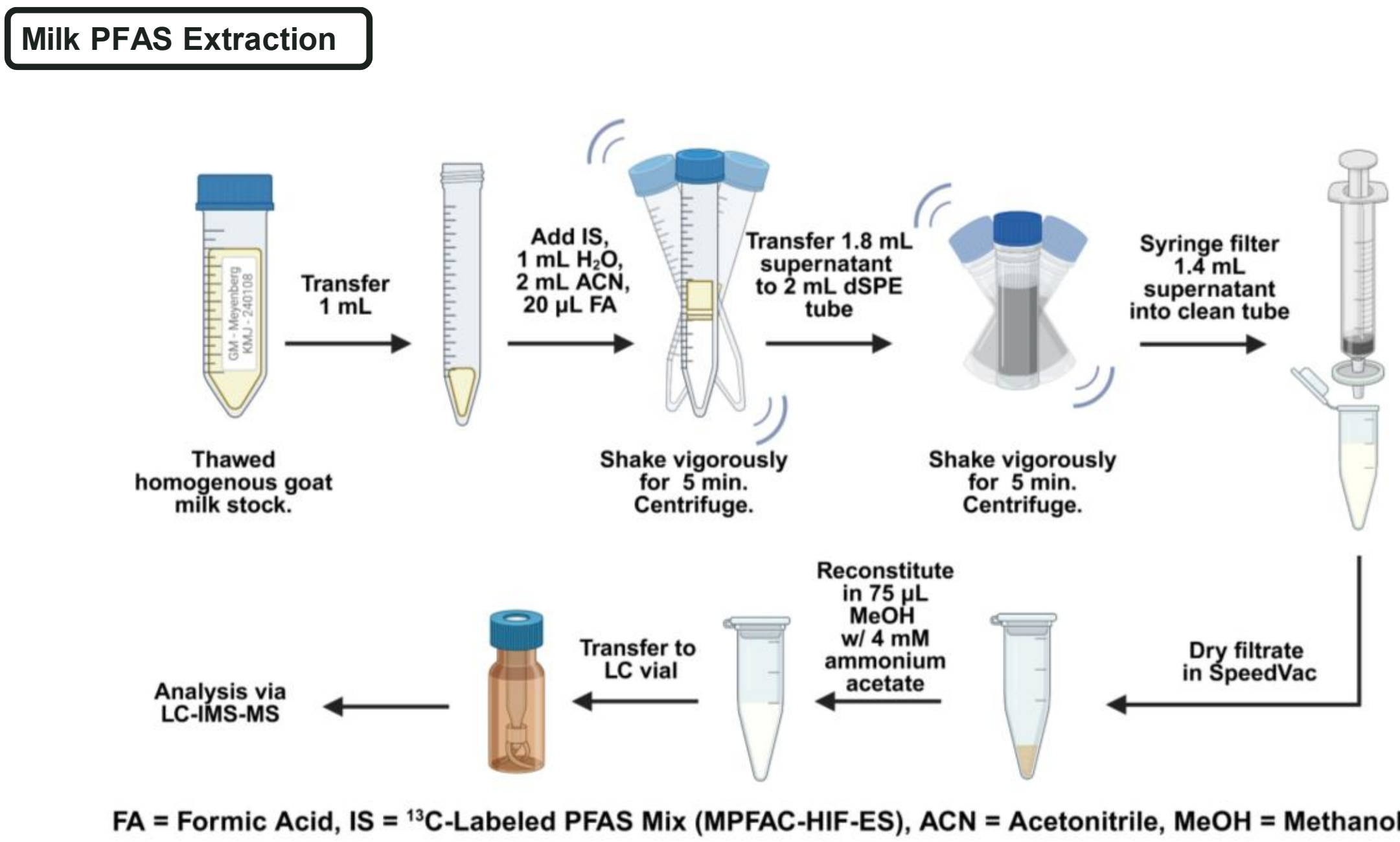

Figure S3. Definitions of LOQ and MDL for quantitative and suspect screening analyses.

For Quantified Analytes	
**Limit of Quantitation (LOQ)**	• LOQ was defined as the lowest point on the calibration curve which has a maximum CV (RSD) of 20%
**Method Detection Limit (MDL)**	• This study follows recommendation from the EPA based on method blanks (EPA 821-R-16-006, 2016)<br>• MB is defined as the **calculated concentration** of PFAS species identified in goat milk or charcoal stripped fetal bovine serum samples spiked with internal standard and taken through the extraction process.<br>1. If none of the method blanks yield a peak area >0 for an individual analyte, MDL is defined as the lowest detectable concentration following data processing and demultiplexing.<br>2. If detected in all of the method blanks, calculate MDL as the X_{MB} + 3*SD_{MB}, where X is the average concentration of the method blanks.

For Suspect Screening	
**Method Detection Limit (MDL)**	• This study follows recommendation from the EPA based on method blanks (EPA 821-R-16-006, 2016)<br>• MB is defined as the **peak area** of PFAS species identified in goat milk or charcoal stripped fetal bovine serum samples spiked with internal standard and taken through the extraction process.<br>1. If none of the method blanks yield a peak area >0 for an individual analyte, MDL is defined as the lowest detectable peak area following data processing and demultiplexing.<br>2. If detected in all of the method blanks, calculate as the X_{MB} + 3*SD_{MB}, where X is the average peak area of the method blanks.

Figure S4. Determination of quantitation accuracy to NIST SRM 1950 (Human Plasma) & 1954 (Fortified Human Breastmilk). For NIST SRM samples, error bars represent expanded uncertainty listed in the associated. For values obtained from this analysis, error bars represent one standard deviation above and below the average measurement. * indicates that the NIST SRM reported value is below the limit of quantitation achieved in this study.

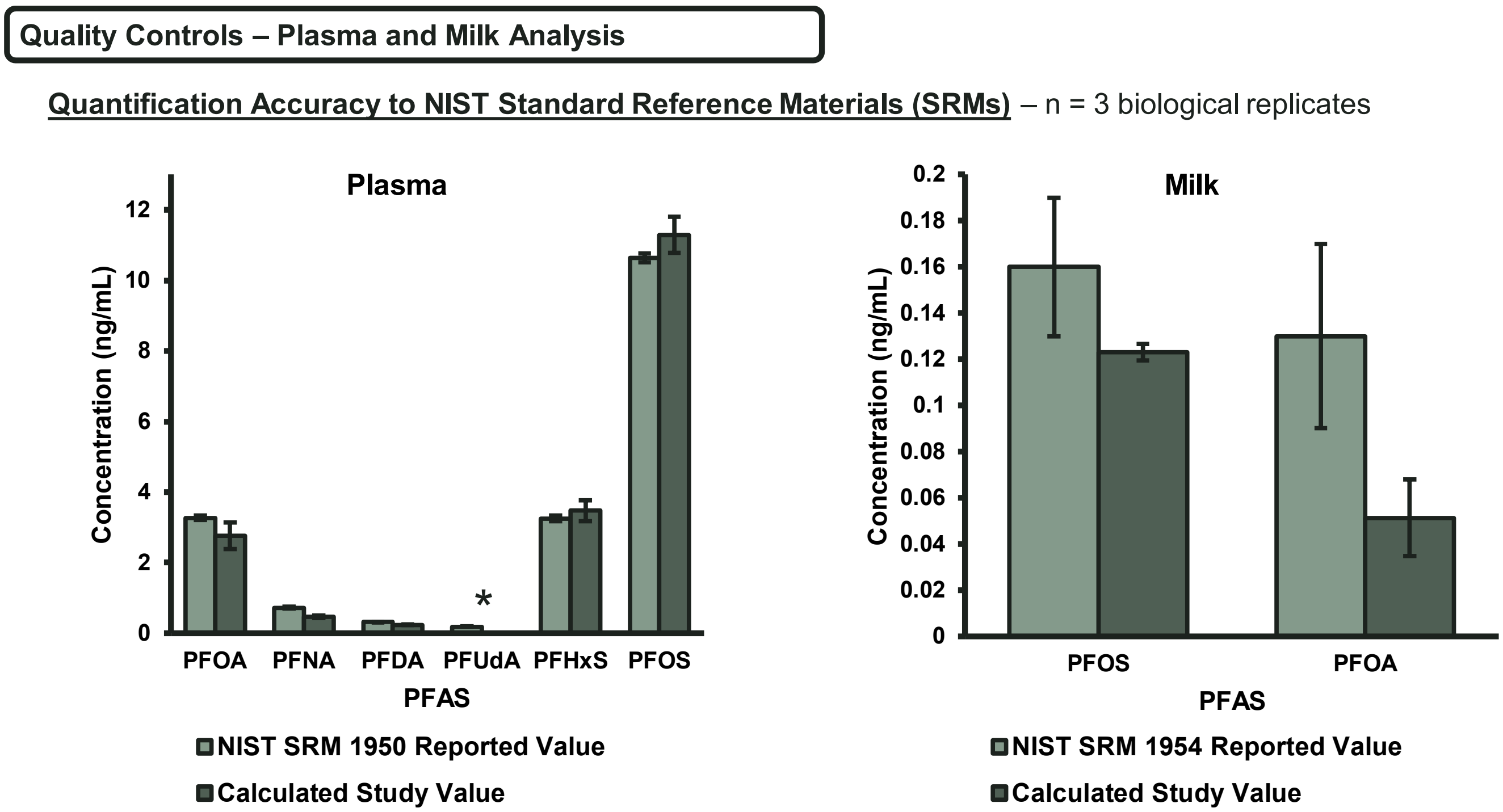

Figure S5. Below are the guidelines for imputing quantitative data presented in this study. Imputation is used to account for values in the dataset below the MDL and between the MDL and LOQ. This scheme is based on the MDL for each analyte in the measurement's respective matrix.

**Imputation Scheme**	
**If Raw Value is…**	**Impute as:**
**Measured Concentration < MDL**	$Imputed\ Value = \frac{MDL}{\sqrt{2}}$
**MDL ≤ Measured Concentration < LOQ**	$Imputed\ Value = MDL + \frac{MDL}{\sqrt{2}}$
**LOQ ≤ Measured Concentration**	$Imputed\ Value = Measured\ Concentration + \frac{MDL}{\sqrt{2}}$

Figure S6. Associations between PFAS concentrations and maternal adiposity, parturition, and age were evaluated, but no substantial correlations were identified, as seen by R^2 values below 0.5.

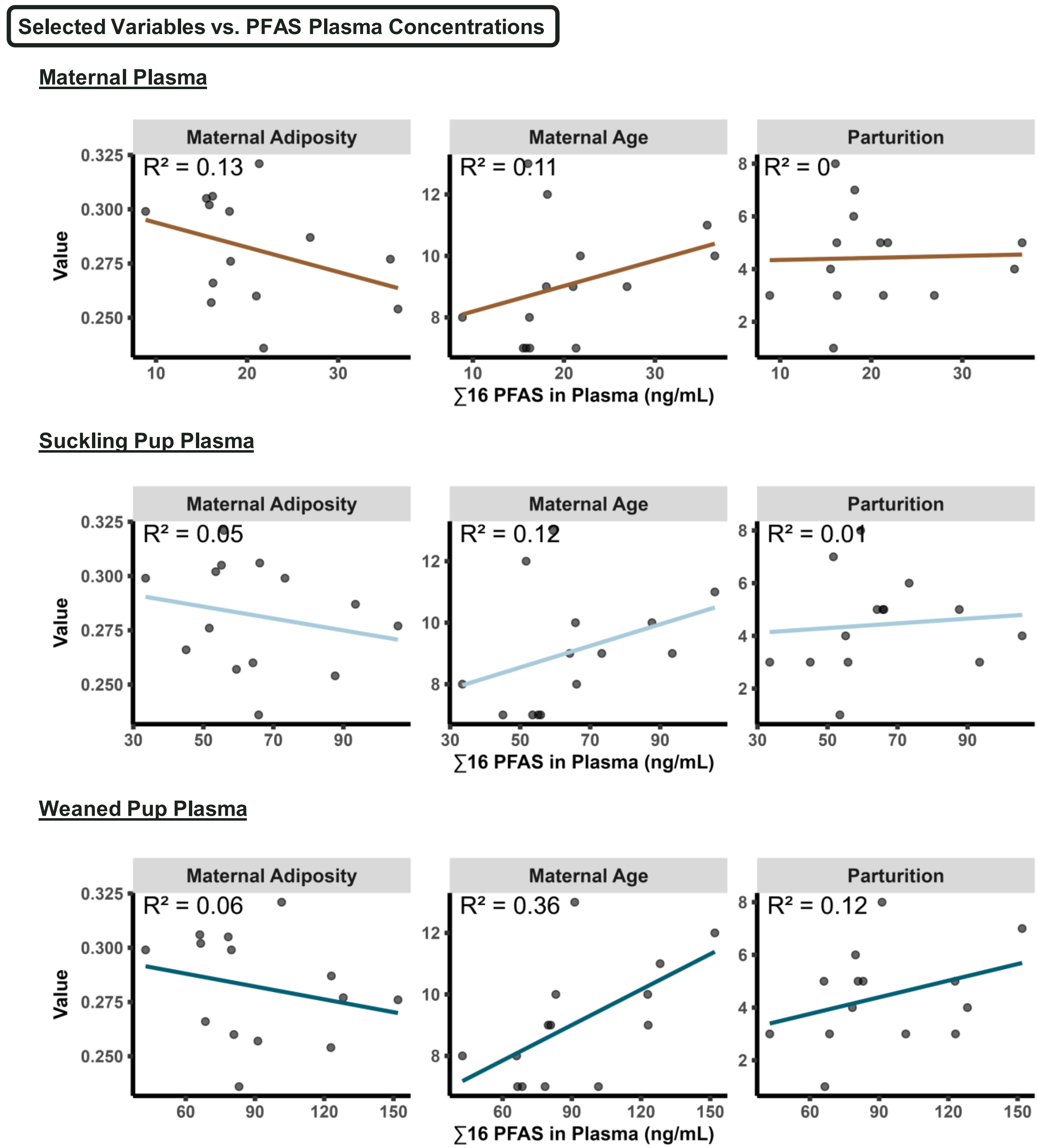

Figure S7. Additional information for PCA of imputed and log_2-transformed plasma PFAS concentrations, with grouping by life stage.

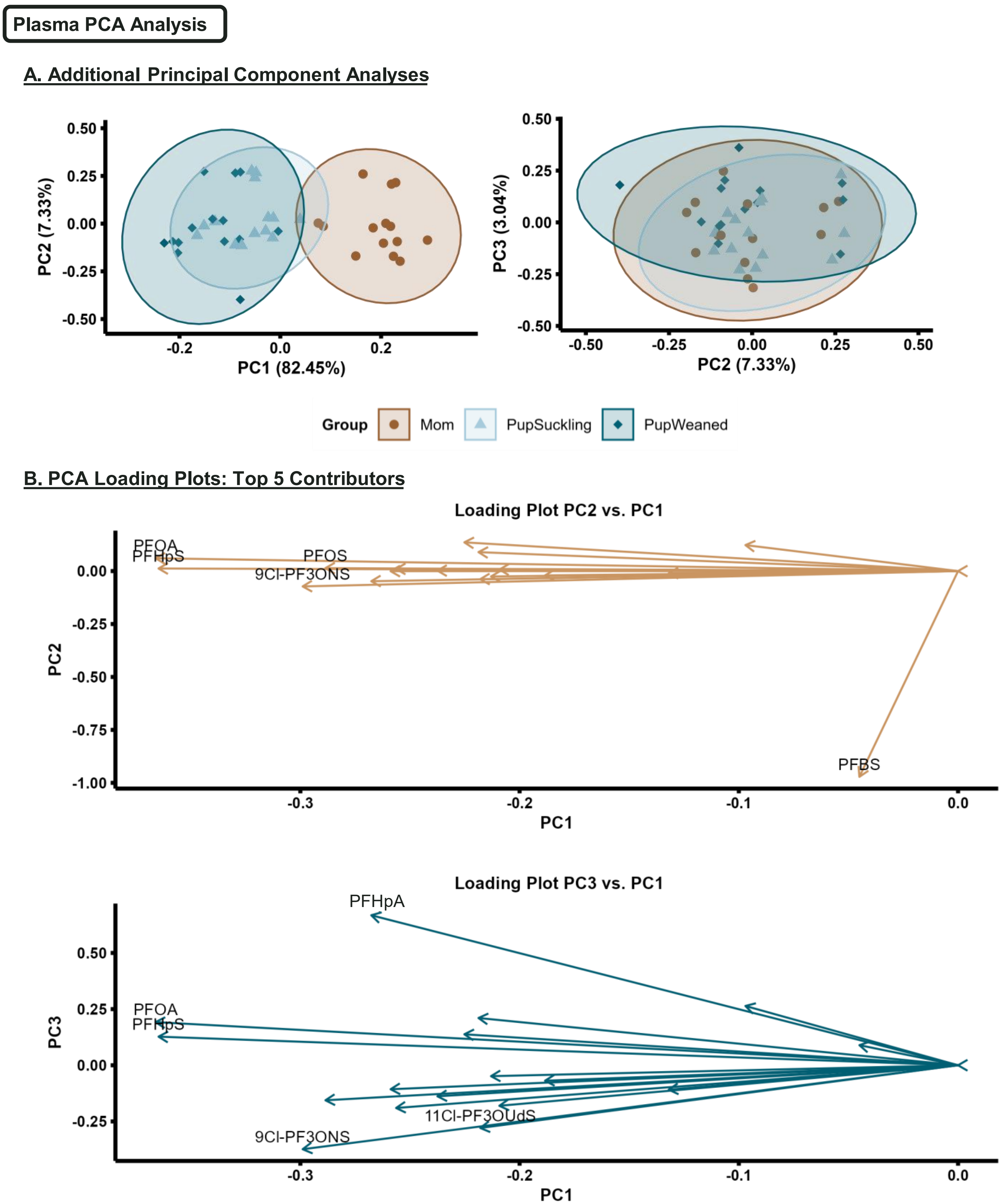

Figure S8. Pairwise comparisons for each PFAS with at least one value above the LOQ by group. Concentrations shown are imputed, log_2 transformed and used to compute statistics.

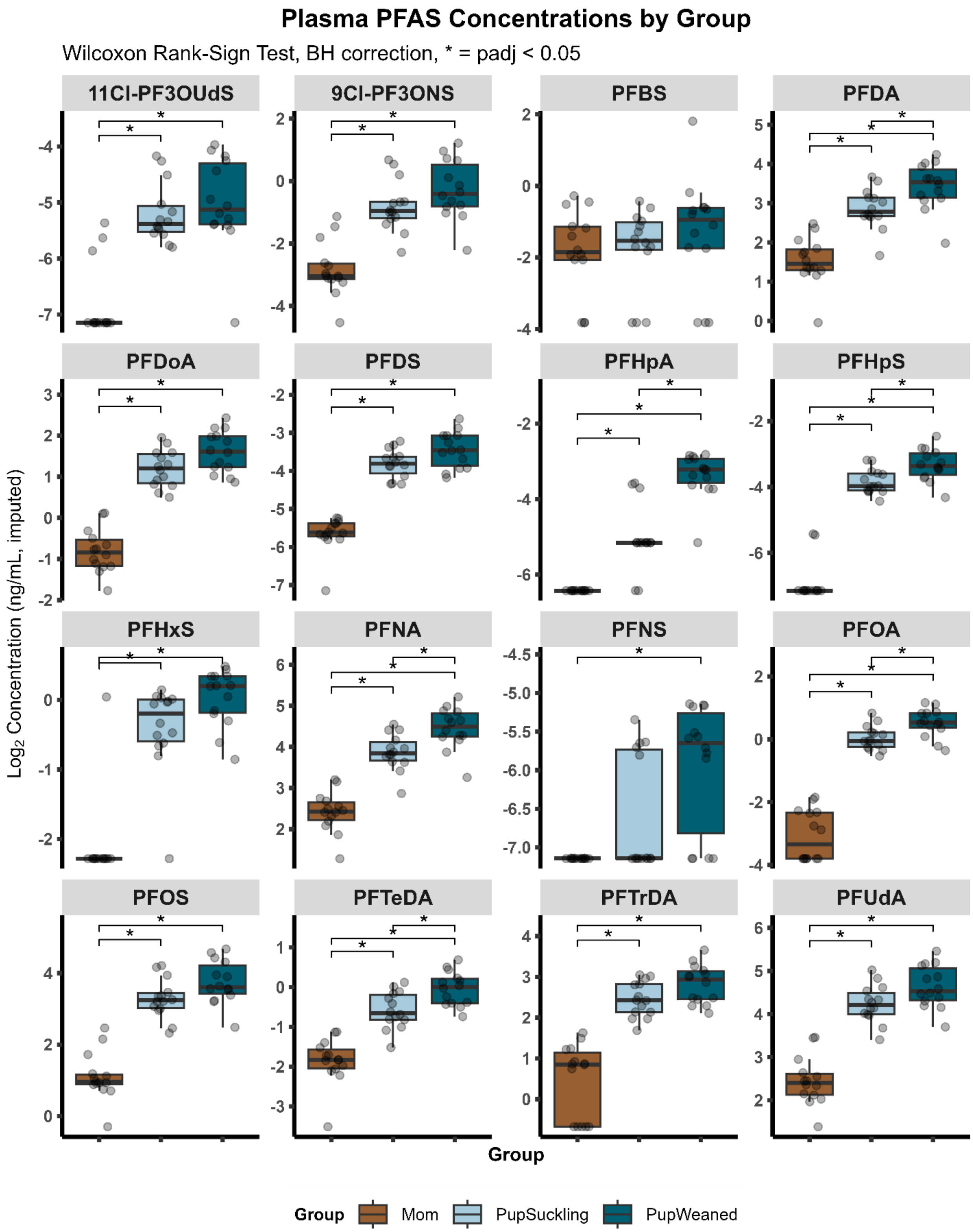

Figure S9. Example calculations for estimates of total dietary PFAS exposure attributable to milk consumed during the lactation period and allometric scaling of TWI for NES.

Sample Calculation of Weekly Dietary PFAS Intake

According to Ortiz et al. (1984), an elephant seal will consume, on average 137.7 kg of milk during the ~4-week lactational period, or 34.4 kg milk/week. Our study found the average milk density to be 952.5 mg/mL of milk, and the average sum PFAS concentration (∑12) to be 5.874 ng PFAS/mL of milk. The average weight of suckling pups whose mother's milk is represented by this data was 123 kg. Below are sample calculations for estimating the weekly dietary intake of PFAS for an elephant seal pup due to lactation.

∑12 PFAS

$$\frac{5.874\ ng\ PFAS}{1\ mL\ milk} \times \frac{1\ mL\ milk}{952.5\ mg\ milk} \times \frac{1\ \times 10^6 mg}{1\ kg} \times \frac{34.4\ kg\ milk}{1\ week} \approx 212{,}000\ ng\ PFAS/week$$

$$\approx \frac{212{,}000\ ng\ PFAS/week}{123\ kg} \approx 1{,}730 \frac{ng\ PFAS}{kg\ bw/\ week}$$

∑(PFOS + PFNA + PFOA + PFHxS)

$$\frac{2.303\ ng\ PFAS}{1\ mL\ milk} \times \frac{1\ mL\ milk}{952.5\ mg\ milk} \times \frac{1\ \times 10^6 mg}{1\ kg} \times \frac{34.4\ kg\ milk}{1\ week} \approx 83{,}200\ ng\ PFAS/week$$

$$\approx \frac{83{,}200\ ng\ PFAS/week}{123\ kg} \approx 676 \frac{ng\ PFAS}{kg\ bw/\ week}$$

Allometric Scaling Using Bodyweight$^{3/4}$

Allometric Scaling Equation: $\boldsymbol{ES\ Dose = Human\ Dose\ \left(\frac{W_d}{W_h}\right)^{3/4}}$

$\boldsymbol{W_h} = Average\ Adult\ Human\ Weight\ (United\ States) = \ 84\ kg$

$\boldsymbol{W_d} = Average\ Suckling\ Pup\ Weight = 123\ kg$

EFSA

$$\boldsymbol{EFSA}\ Weekly\ Intake\ Recommendation = 4.4 \frac{ng}{kg\ bw/week}$$

$$Human\ Dose = \ 4.4 \frac{ng}{kg\ bw/week} \times 84\ kg\ bw = 369.9\ ng/week$$

$$ES\ Pup\ Dose = 369.9 \frac{ng}{week} \times \left(\frac{123\ kg}{84\ kg}\right)^{3/4} \approx 490\ ng/week$$

$$ES\ Pup\ TWI = \frac{490\ ng}{week} \div 123\ \mathrm{kg} \approx 4.0\ \mathrm{ng\ PFAS/kg\ bw/week}$$

Figure S10. Plasma vs. milk concentrations (ng/mL) for each PFAS commonly quantified between both matrices (n = 9 individuals per group). Generally, as milk concentrations increase, as do plasma concentrations for each group.

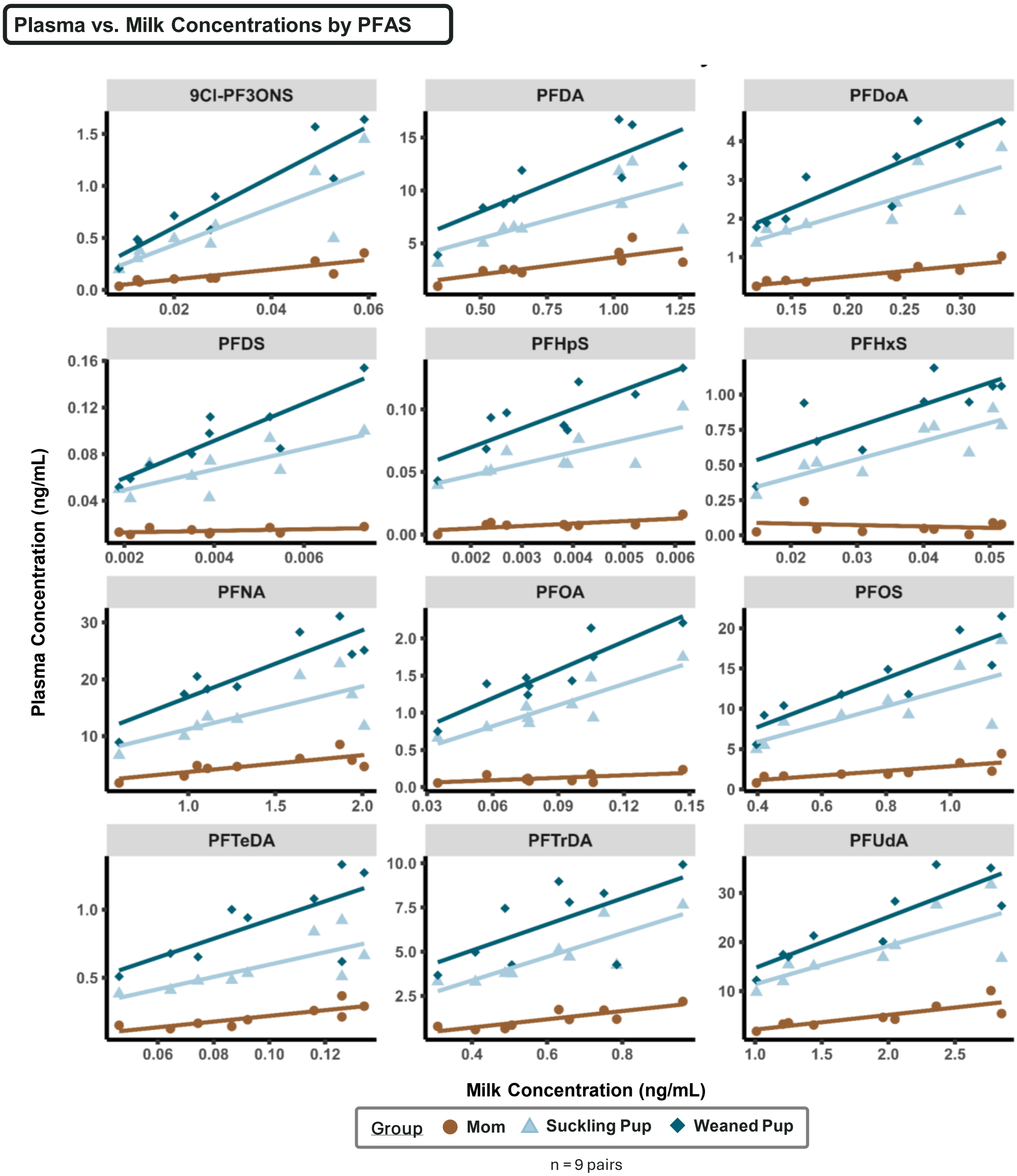

Figure S11. Full intersection (Upset plot) analysis, including two PFAS with Level 3 Confidence Identification: trifluoromethyl sulfonic acid (TFMS) and unsaturated PFOS (U-PFOS).

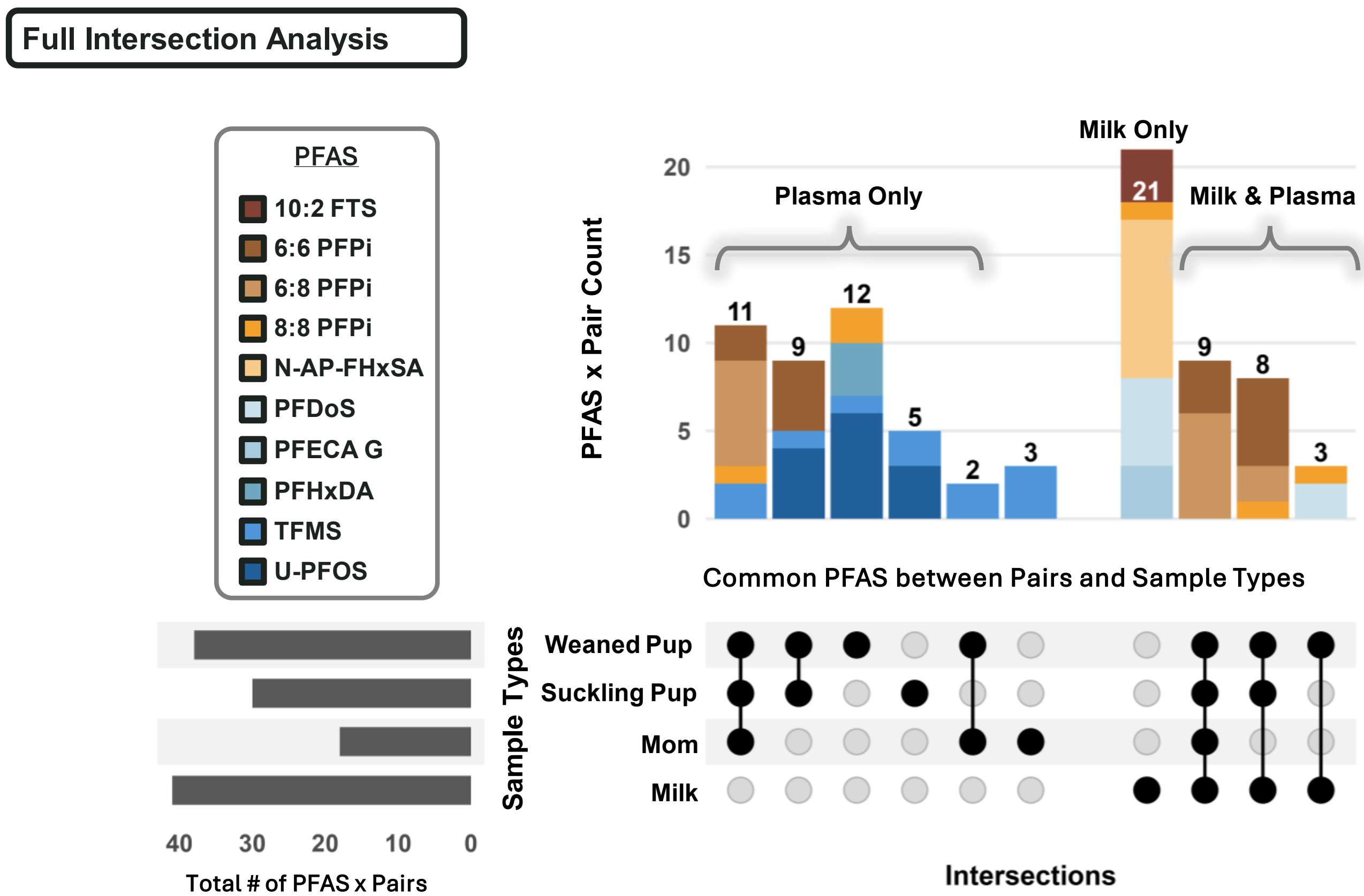

Supplement: Supplementary file 1 [file eh5c00782_si_001.pdf]
